# Simultaneous Bilateral Quadriceps Tendon Rupture following Long-Term Low-Dose Nasal Corticosteroid Application

**DOI:** 10.1155/2013/657845

**Published:** 2013-07-31

**Authors:** Mohamed Omar, Philipp Haas, Max Ettinger, Christian Krettek, Maximilian Petri

**Affiliations:** Trauma Department, Hannover Medical School, Carl-Neuberg-Straße 1, 30625 Hannover, Germany

## Abstract

Simultaneous bilateral quadriceps tendon rupture is a very rare injury, which was previously only described in slightly more than 100 cases in the English literature. Occurrence after minor trauma is predominantly associated with certain medical conditions including chronic diseases and long-term use of certain drugs. We report the case of a 61-year-old healthy patient who sustained a simultaneous bilateral quadriceps tendon rupture following minor trauma. Medical history was completely clear except of a long-term nasal corticosteroid medication due to allergic rhinitis.

## 1. Introduction

Quadriceps tendon rupture predominantly occurs after major trauma in younger patients or as the result of tendon weakening due to obesity and arteriosclerosis-induced fibrotic changes or previous injury [1].

Simultaneous bilateral quadriceps tendons rupture is a very rare injury. Since the first description by Steiner and Plamer [2], only slightly more than 100 cases were reported in the English literature. It mostly occurs in patients with chronic diseases including chronic renal failures [3], hyperparathyroidism [1], gout, systemic lupus erythematosus [4], and long-term use of drugs such as quinolones, statins [5], and anabolic steroids [6].

Corticosteroid application is known to produce a variety of adverse effects including the induction of apoptosis of tendon cells resulting in partial or complete rupturing [7]. This effect is very well described following local and systemic medication [8, 9]. However, adverse effects resulting in quadriceps tendon rupture following nasal application of corticosteroids have not been found yet [10]. According to Blanco et al.[11], singular cases of Achilles tendon ruptures and tendinopathies after topical application (e.g. inhalation, nasal, and cutaneous) were reported.

We report the case of a 61-year-old healthy man who sustained simultaneous bilateral quadriceps tendons rupture after a minor trauma. Long-term low-dose nasal application of a corticosteroid due to an allergic rhinitis was identified as exclusive predisposing factor.

## 2. Case Presentation

A 61-year-old man was admitted to our emergency department who stumbled and consequently fell on both knees. The patient heard loud cracks as he touched the ground. Immediately after the trauma, he was not able to bear weight and collapsed due to giving way of both knees. 

The patient was retired and did not perform heavy labour throughout his life. He was regularly performing sportive activities including swimming and cycling. Medical history was completely clear except of daily use of a nasal corticosteroid (mometasone-17-2-furoate) in a concentration of 300 *μ*m per day for two years due to allergic rhinitis. The patient declined the use of any other medications, especially those that are known to be associated to tendon ruptures. There was no history of tendon or joint pathology before steroid treatment.

On physical examination, there was oedematous swelling and hematoma in the suprapatellar region as well as intra-articular effusion of both knees. There was no skin affection. Palpation did not reveal osseous disruption of the patella. In the suprapatellar region of both quadriceps tendons, soft tenderness was palpable. Patellae showed increased instability for lateral, medial, and distal, but not for cranial, shifting indicating intact patellar ligaments. Quadriceps contraction did not result in movement of the patellae. The patient was unable to actively extend his legs or to lift the extended legs. There was full range of motion and muscle strength for active flexion of the knees. Neurologic and neurovascular examinations were normal. 

**Figure 1:**
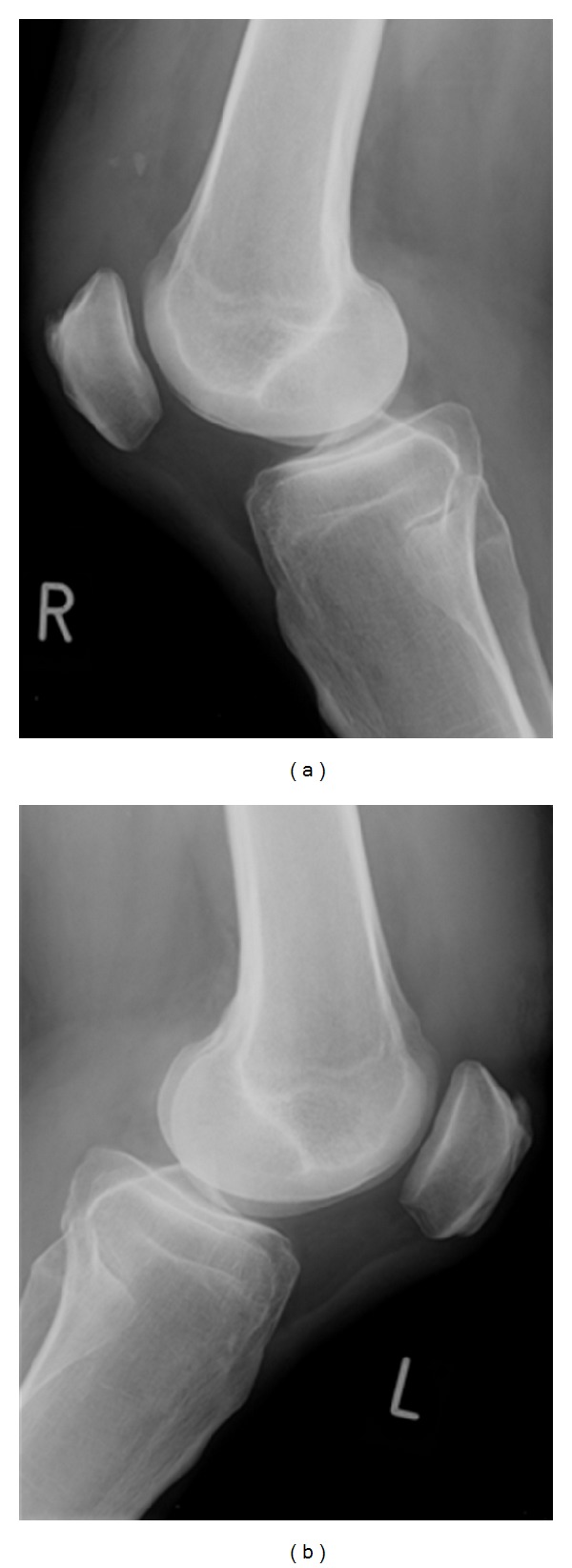
Plain radiographies show low riding of both patellae and signs of suprapatellar ossification of the right knee.

Plain radiography showed low riding of both patellae and signs of suprapatellar ossification of the right knee indicating pretraumatic degeneration as illustrated in Figure 1.

**Figure 2:**
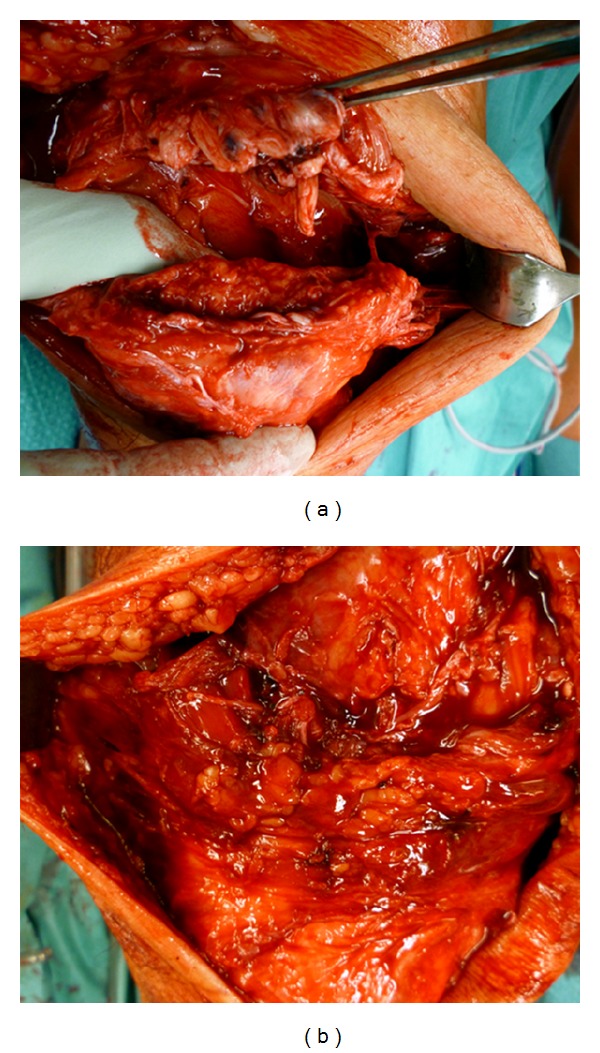
Intraoperative pictures of the ruptured quadriceps tendons. On the right side (a), the rupture is directly at the insertion site of the tendon to the suprapatellar aspect while a intratendinous rupture is apparent on the left side (b).

In sonographic examination, a complete disruption of the quadriceps tendon at the patella insertion sites was revealed. The patient was operated two days after injury. Ruptured tendons were exposed through a median approach. A full-thickness rupture was observed at the insertion site of the superior patellar aspect on the right leg as shown in Figure 2. The left side showed a full-thickness intratendinous tear of the quadriceps tendon 3 cm proximal to its insertion point at the patella.

**Figure 3:**
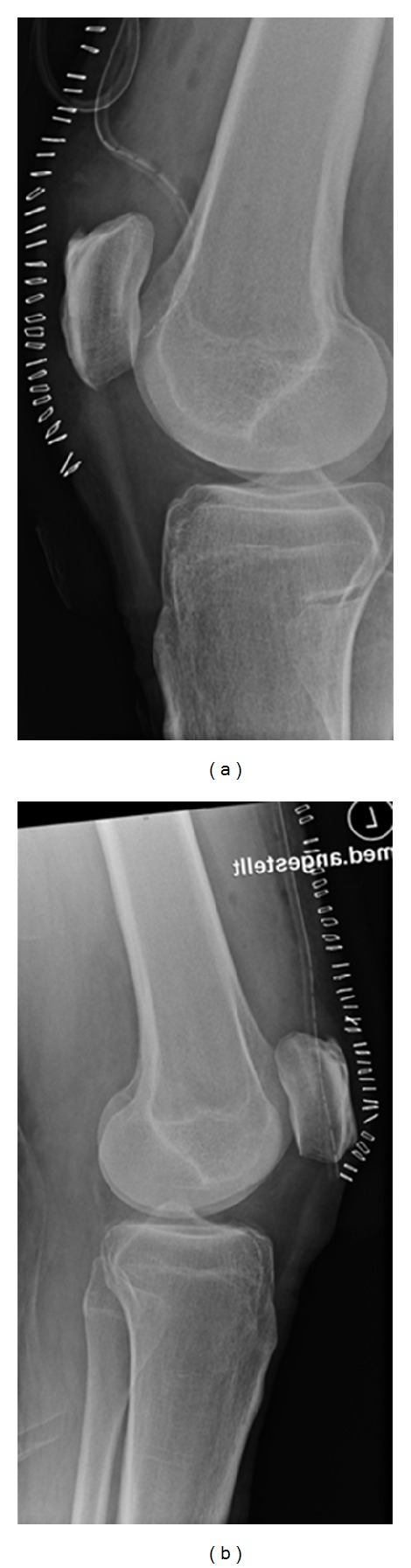
Postoperative radiographies demonstrate regular positioning of the patellae.

The stumps were trimmed and stitched through transosseous sutures using a 2.5 mm drill and a fibre wire with a double row Bunnell technique. Postoperative radiography showed regular positioning of the patellae as illustrated in Figure 3. 

**Figure 4:**
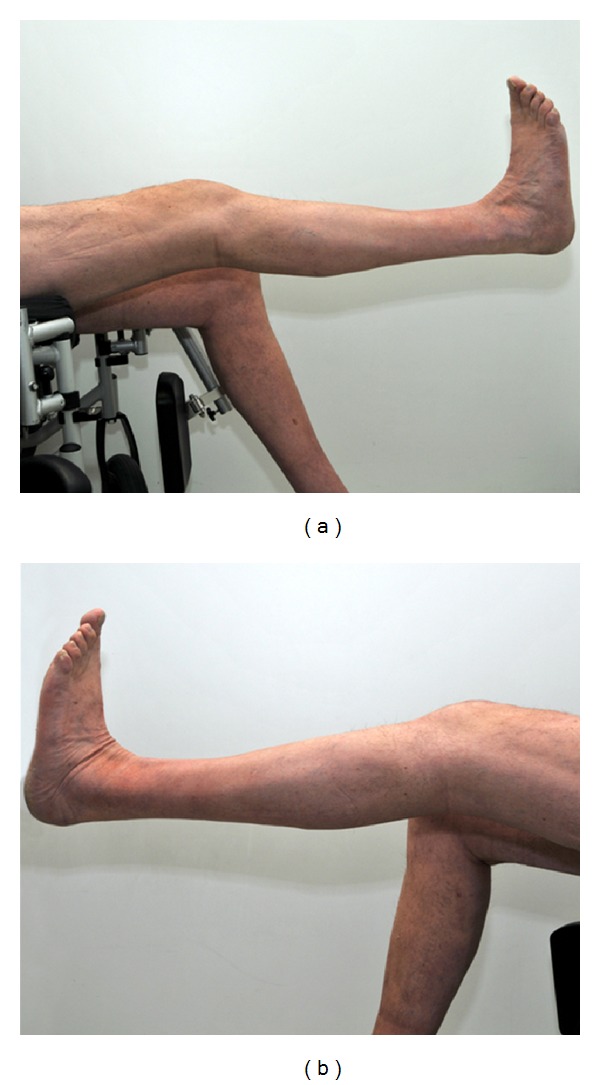
Six weeks following tendon suturing, the patient was able to extend the knee actively. However, there was an extension lag of 10° at the right side (a) and 15° at the left side (b).

Using a range-of-motion brace, knee flexion was postoperatively limited to 60 degrees for three weeks following another period of three weeks with flexion limitation of 90 degrees. The patient was not allowed to perform weight bearing for the time of knee flexion limitation. After 6 weeks, the patient was able to do full weight bearing. There was an extension lag of 10° at the righe side and 15° at the left side as shown in Figure 4.

## 3. Discussion

The quadriceps femoris muscle is the largest muscle in humans and primary performs extension of the knee. The muscle inserts to the tibial tuberosity through the patella and patella ligament. The quadriceps tendon is attached to the superior aspect of the patella. It can resist considerable loads. It could be demonstrated that approximately 50% of a healthy tendon's tissues have to be severed to result in rupturing. Quadriceps tendon rupture can occur by direct or indirect mechanisms. Most traumas involve eccentric contraction while weight bearing in a partially flexed position of the knee [12]. 

Typical history, trauma mechanism, and evaluation of the risk factors can lead to the diagnosis. The trias of symptoms with painful swelling, palpable suprapatellar gap, and the inability to actively extend the knee is not obligatory and only found in about 60% of patients [13]. Plain radiographs reveal only indirect signs of the lesion including intraarticular swelling, shadow in the tendon line, patellar spurs at the tendons insertion, and low riding and forward tilting of the patella. In case of systemic diseases or degeneration, suprapatellar calcifications due to bony transformation can be observed [5]. Ultrasound and magnetic resonance imaging can directly prove the rupture as they visualize the soft tissue.

Quadriceps tendon rupture is a common injury of the knee after major trauma in younger patients. In elderly patients, rupture is the result of tendon weakening due to obesity and arteriosclerosis-induced fibrotic changes or previous injury [1]. Bilateral and simultaneous rupture is a rarity. Since the first description by Steiner and Plamer [2], only slightly more than 100 cases were reported in the English literature. Bilateral ruptures are generally associated with chronic diseases including chronic renal failures [3], hyperparathyroidism, gout, systemic lupus erythematosus [4], or long-term use of drugs such as quinolones, statins [14], and anabolic steroids [6].

Tendon rupture following corticosteroid medication is very well described. Systemic and local application of corticosteroids is known to generate a variety of adverse effects. These include growth inhibition induced by hypothalamus-pituitary-adrenal (HPA) axis suppression, decreased bone mineral density, myopathy, cataract, glaucoma, hypertension, hyperglycemia, and skin affections [15]. 

The occurrence of tendon ruptures and impaired tendon healing following systemic or local corticosteroid usage have been described previously. Newnham et al. [9] and Kotnis et al. [8] reported atraumatic ruptures of Achilles tendon in patients with respiratory diseases treated with systemic corticosteroids. The relation between local corticosteroid application and tendon rupture could be demonstrated by Nguyen and Jones [16] who observed the rupture of both the abductor pollicis longus and extensor pollicis brevis tendons after repetitive steroid injection for treatment of de Quervain tenosynovitis. Although the mechanism is not elucidated in detail, it is assumed that steroids suppress the repair of partially ruptured or degenerated tendons. Therefore, minor trauma can easily lead to complete rupture of the already damaged tendon. These findings are supported by in vitro observations of Hossain et al. [7] who could demonstrate that increasing concentrations of corticosteroids inhibit the proliferation and induce apoptosis of cultivated canine tendon cells. 

In our case, there was no history for local or systemic corticosteroid usage. Moreover, no joint or tendon pathologies have been reported before the accident. The suprapatellar calcification in the right knee was the only evidence for pretraumatic degeneration as a possible result of a chronic corticosteroid medication. Corticosteroids are known to exert their negative effect on bone metabolism by altering both calcium homeostasis and sex hormone production [17]. Suprapatellar calcifications are not exclusive for corticosteroid medication, but the occurrence of calcifications may be a sign of corticosteroid associated adverse effects. Apart from that, the patient did not show any noticeable medical conditions. He was a healthy 61-year-old man who regularly performed endurance activities like cycling and swimming known not to be stressful for tendons. He never had any tendinopathies or injuries of the musculoskeletal system. The patient sustained bilateral quadriceps rupture after a minor trauma as he stumbled and fell on both knees. As discussed earlier, the trauma mechanism is not typical for quadriceps tendon rupture. As the incidence of bilateral quadriceps tendon ruptures without any underlying risk factors is very low [18], we assumed that the corticosteroid medication could play a certain role.

The long-term use of nasal mometasone in a concentration of 300 *μ*g per day for two years appeared to be the exclusive risk factor. However, as reviewed in detail by Sastre and Mosges [10], nasal steroid application has been shown to be safe and in first line leads to local adverse effects including nasal irritation and epistaxis. In many studies, it could be demonstrated that nasal corticosteroid does not alter the concentration of blood cortisol level. However, certain amounts reach systemic circulation, and adverse effects are therefore considerable. Although rarely reported, there were singular cases of tendinopathies after topical (i.e., ocular, cutaneous, and nasal) corticosteroid use [11]. These cases predominantly consisted of Achilles tendon ruptures and in a minority of cases of tendinitis or an unspecified tendon disorder. Tendinopathies or even ruptures of the quadriceps tendons after topical corticosteroid medication have not been described yet. 

Although nasal application of corticosteroids seems to be safe, in a minority of cases adverse systemic effects have been reported. This is the first case to demonstrate a possible relation between bilateral quadriceps tendon ruptures after a minor trauma and long-term use of nasal corticosteroids. Further investigations are required to prove this assumption. However, as corticosteroids in general are known to generate adverse effects on tendons, topic application should be taken into consideration as potential risk factor for tendinopathies.
